# Occurrence of *Aelurostrongylus abstrusus* in domestic cats in Vilhena, Rondônia, Brazil

**DOI:** 10.1590/S1984-29612022053

**Published:** 2022-09-30

**Authors:** Enny Caroline Ferreira Farago, Acácio Duarte Pacheco, Patrícia Fernandes Nunes da Silva Malavazi, Mariasole Colombo, Simone Morelli, Angela Di Cesare, Soraia Figueiredo de Souza

**Affiliations:** 1 Centro de Ciências Biológicas e da Natureza, Universidade Federal do Acre, Rio Branco, AC, Brasil; 2 Facoltà di Medicina Veterinaria, Università degli Studi di Teramo, Teramo, Abruzzo, Italia

**Keywords:** Aelurostrongylus abstrusus, cat, Brazil, Aelurostrongylus abstrusus, gatos, Brasil

## Abstract

Aelurostrongylosis, caused by the metastrongylid nematode *Aelurostrongylus abstrusus*, is an underestimated feline respiratory disease. Cats infected by *A. abstrusus* may show subclinical to severe clinical signs. *Aelurostrongylus abstrusus* has a worldwide distribution. Nevertheless, studies on this parasite in Brazil are scarce, and most have been conducted in the southern regions. This study investigated the occurrence of *A. abstrusus* in cats in Vilhena, Rondônia, Brazil, from April 2020 to February 2021. Three consecutive individual fecal samples from 101 cats were examined using Baermann and Hoffman tests. Two cats (1.98%) scored positive for *A. abstrusus*, one with the Baermann examination, and one with the Hoffman technique. No other lungworms were retrieved. The clinicopathological and epizootiological implications are described and discussed.

## Resumo

A aeluroestrongilose, causada pelo nematoide metastrongilídeo *Aelurostrongylus abstrusus,* é uma doença respiratória subestimada. Os gatos infectados pelo *A. abstrusus* podem ser assintomáticos ou apresentarem sinais graves. O *A. abstrusus* possui distribuição cosmopolita. Apesar disso, estudos sobre esse parasita no Brasil são escassos, e a maioria deles são realizados na região Sul. Este estudo tem como objetivo avaliar a ocorrência de *A. abstrusus* em gatos de Vilhena, Rondônia, Brasil. De abril de 2020 a fevereiro de 2021, foram avaliadas três amostras consecutivas de fezes de 101 felinos, utilizando-se as técnicas de Baermann e de a Hoffman. Dois gatos (1,98%) foram positivos para *A. abstrusus*, um pela técnica de Baermann e outro pela técnica de Hoffman. Não foram encontrados outros parasitas pulmonares. Os achados clínicos dos gatos positivos foram descritos, e os resultados deste estudo discutidos sob aspectos clínicos e epidemiológicos.

*Aelurostrongylus abstrusus* is a worldwide nematode that is distributed worldwide and is considered the most common feline lugworm (Lima et al., 2021; Morelli et al., 2021). The distribution of *A. abstrusus* is well-known in Europe, however, reports from Brazil are limited (Lima et al., 2021; Morelli et al., 2021).

The life cycle of *A. abstrusus* is indirect, with slugs and snails, such as *Lissachatina fulica* (Lima & Guilherme, 2018), *Biomphalaria glabrata* (Zottler & Schnyder, 2016), and *Rumina decollata* (Cardillo et al., 2014) as intermediate hosts in South America. Adult stages inhabit the alveoli, alveolar ducts, and bronchioles of domestic cats where they lay eggs. Hatched first-stage larvae (L1) move up to the pharynx, and are then swallowed and released into the environment with feces. L1 then develops into infective third-stage larvae (L3) in intermediate hosts. Infection in domestic cats occurs through the ingestion of slugs, snails, or paratenic hosts, such as rodents, lizards, and birds, harboring infective L3 (Elsheikha et al., 2019).

Cat aelurostrongylosis can vary from subclinical and self-limiting to severe. Clinical signs include coughing, sneezing, tachypnea, open-mouth breathing, and dyspnea. Non-specific manifestations such as prostration and weight loss may occur. In most severe infections, pleural effusion, pyothorax, and death have been reported (Gueldner et al., 2019). The disease can be more severe in cases of high worm burden, young cats, or animals affected by other concomitant diseases (Elsheikha et al., 2016).

The radiographic findings in cats infected with *A. abstrusus* are not specific, with mixed patterns, such as alveolar, interstitial (either nodular or understructured), and vascular patterns, overlapping with other respiratory pathological conditions of cats (e.g., feline bronchial disease/asthma, infectious pneumonia). Laboratory findings of aelurostrongylosis are highly non-specific, that is, leukocytosis with eosinophilia and mild anemia, occasional lymphocytosis, monocytosis, and basophilia (Morelli et al., 2021).

Data on the prevalence of cat aelurostrongylosis are highly variable, based on the differences in the populations studied, number of samples *per* study, geographic areas, and diagnostic techniques used (Carvalho, 2017). In South America, feline lungworm infections have not received a high level of attention from veterinary practitioners and researchers. Consequently, aelurostrongylosis may be underestimated (Penagos-Tabares et al., 2018). In Brazil, the prevalence of *A. abstrusus* in cats ranges from 1.3% (Ramos et al., 2013) to 38.1% (Kusma et al., 2015). In western Amazonia (i.e., Rio Branco, Acre), the occurrence of the parasite has been demonstrated only recently (Lima et al., 2021). Specifically, the L1 of *A. abstrusus* has been retrieved in 2.5% of the cats examined, and the first molecular unequivocal identification of the parasite in a cat in Brazil has been provided (Lima et al. al., 2021). In the northern region of the country, the occurrence of the parasite has also been described in intermediate hosts, with infection rates of 40% in African giant snails (*Achatina fulica*) from Rio Branco (AC) (Lima & Guilherme, 2018). In Rondônia, there are no previous reports of *A. abstrusus* infections in domestic or wild cats, although the presence of *A. fulica* has been documented Colorado do Oeste, 86 km from the municipality of Vilhena (Rondônia) (Alves et al., 2017). Thus, this study aimed to investigate the occurrence of *A. abstrusus* in this area, as constant epizootiological surveillance is pivotal for diagnostic, therapeutic, and control measures.

This study was submitted to the Ethics Committee on the Use of Animals (process 23107.016278/2009-24), and was approved under protocol nº 21/2019.

The study was conducted in a veterinary clinic located in the city of Vilhena, Rondônia. Vilhena is a Brazilian municipality in the northern region of the country that belongs to the state of Rondônia, latitude: 12° 44' 3” South, longitude: 60° 8' 41” West. According to the Brazilian Institute of Geography and Statistics it is the fourth most populous municipality in the state, with an estimated population of 102.211 people. It has a typical hot and humid climate in the Amazon region, with a rainy season from September to May (IBGE, 2021).

Using a convenience sample, the survey included 101 owned cats or living in a shelter from April 2020 to February 2021, brought to a veterinary clinic in Vilhena for any reason such as routine vaccination or veterinary consultation. The exclusion criteria were cats under 3 months of age. The owners signed informed consent forms. Data on signalment, anamnesis, antiparasitic treatment, access to the outdoor environment, hunting habits, and the presence of slugs or snails in the living environment were collected for each cat. Cats were subjected to physical examination and clinical alterations were recorded.

For each cat, fecal samples were collected by the owners for three consecutive days, directly from the soil or litter, immediately after elimination. The samples were packed in universal collectors, kept in a Styrofoam box with ice, and immediately transported to the laboratory. The samples were processed using Baermann and Hoffman techniques (Alho et al., 2013). The Baermann technique was performed with 10-15 g of feces wrapped in gauze, suspended in a sedimentation cup filled with warm water, and stored overnight. The sediment was then centrifuged at 1500 RPM (189 g) for 5 min, the supernatant was discarded, and the sediment was assessed. For the Hoffman technique, 4 g of feces were mixed with water and filtered into a sediment cup filled with water, and the sediment was examined after two hours.

L1 were identified according to the published morphological keys (Traversa et al., 2021) and were differentiated from those of other feline lungworms (e.g., *Troglostrongylus brevior*).

Cats that scored positive for lungworms in the copromicoscopic examinations underwent two orthogonal thoracic radiographs to evaluate the presence of pulmonary lesions. Blood samples were collected from the jugular veins. Blood was placed in tubes containing ethylenediamine tetraacetic acid and processed using a hematological analyzer (MINDRAY Vet 2800, Mindray do Brasil, São Paulo, Brazil), according to the manufacturer's recommendations. Differential leukocyte count and platelet count estimations were performed using an optical microscope.

Of the 101 cats examined, 57 (56.43%) were females and 44 (43.56%) were males. A total of 46 (45.54%) females and 32 (31.68%) males were neutered. The ages of the animals ranged from 3 to 17 years. Specifically, 26 (25.74%), 64 (63.36%), and 11 (10.89%) cats were young (≤ 1 year), adult (1-7 years old), and elderly (≥ 7 years), respectively.

Regarding antiparasitic treatment, 29 cats (28.71%) were regularly administered endoparasiticides. The endoparasiticides used most frequently were Basken® (Praziquantel + Oxantel), Petzi Cats® (Praziquantel + Pyrantel), and Drontal Cats® (Praziquantel + Pyrantel). Additionally, 66 (65.34%) cats reported no regular deworming, and six (5.94%) had no history of treatment.

Ninety-seven 97/101 cats (96.04%) were outdoors, but with access limited to the yard, three (2.97%) had access to the streets, and one (0.99%) had been recently rescued from a rural area. Sixty-eight 68/101 (67.32%) owners detected the presence of snails in the environment, 19 (18.81%) did not report the presence of intermediate hosts, and 14 (13,86%) did not know if snails were present.

**Figure 1:**
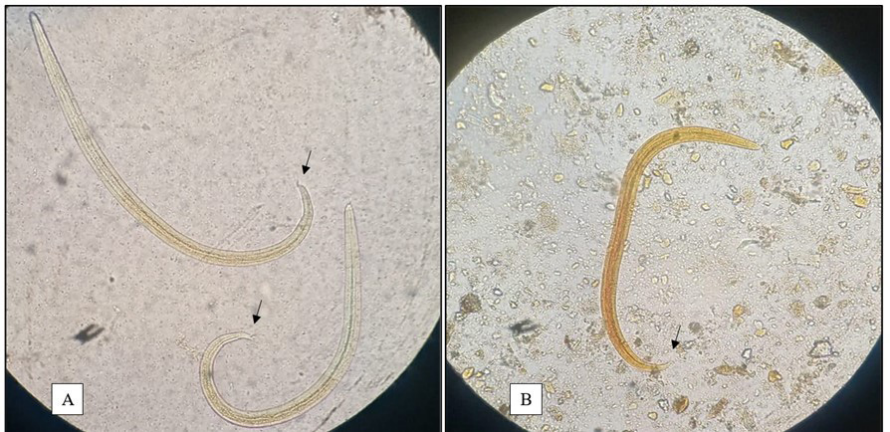
A. First-stage larva of *Aelurostrongylus abstrusus* found in Cat 1 from the city of Vilhena, Rondônia, using the Baermann technique. B. *Aelurostrongylus abstrusus* first-stage larva found in Cat 2 using the Hoffman technique. The head is rounded and has a terminal oral opening, while the tail is kinked (S-shaped) with small finger-like projections. Arrows indicate the posterior end in an “S” shape. 40X magnification.

Overall, two out of 101 cats (1.98%) were infected with *A. abstrusus*. Due to the low occurrence, it was not possible to determine risk factors for aelurostrongylosis in cats. Specifically, one scored positive for lungworm L1 on the Baermann examination (Figure 1A), whereas one scored positive with the Hoffman technique (Figure 1B). L1 were microscopically identified as *A. abstrusus* according to morphological features (i.e., presence of a terminal oral opening and “S” shaped tail with deep dorsal and ventral incisures, and knob-like appendages). Hoffman technique showed *Ancylostoma* spp. and *Strongyloides* spp. eggs in 13.7% (14/102) and 2.9% (3/102) of the cases, respectively. *Isospora* spp. oocysts were detected in 3.9% (4/102) of the cats.

**Figure 2:**
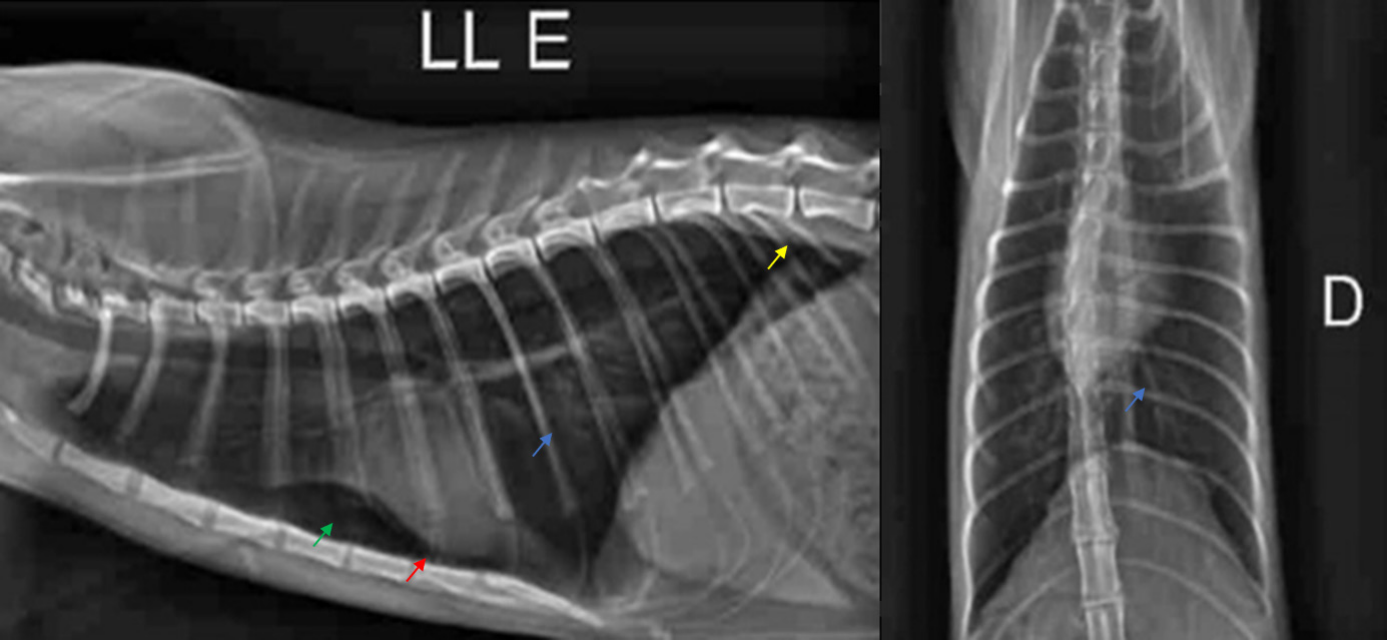
Thoracic radiographs of Cat 1 diagnosed with aelurostrongylosis in Vilhena, RO, Brazil, in the lateral-lateral (A) and ventrodorsal (B) views, showing mild pneumothorax (green narrow), lung hyperinflation (yellow narrow), and a mild bronchial pattern (blue narrow). Mild elevation of the cardiac silhouette next to cardiac apex, raising a radiolucent area between the heart and sternum (red narrow).

The first cat (Cat 1) positive for *A. abstrusus* was a 3-year-old female, recently neutered, mixed breed, weighing 2.7 kg. The animal was subclinically infected and had been recently rescued from a rural area. Larvae were detected in only one sample from the three days of fecal collection. In the complete blood count report, both cats showed stress-induced polycythemia. Cat 1 also presented mild segmented neutropenia and eosinophilia. Mild bronchial pattern, pneumothorax, and lung hyperinflation were observed on radiographic examination of Cat 1. The cardiac silhouette was within the normal limits (Figure 2).

The second cat (Cat 2) infected by *A. abstrusus* was a neutered female mixed breed, approximately one year old. The cat had free access to a yard. The cat did not show any respiratory signs, but was suffering from diarrhea. Cat 2 was positive for *Ancylostoma* sp., which may be related to diarrhea. However, mild diarrhea has been previously described in a feral kitten infected with *A. abstrusus* (Philbey et al., 2014). Blood examination revealed mild leukocytosis and no changes in the WBC differential count. Radiographic examination of the thorax revealed normal findings.

Both cats were treated twice, 15 days apart with fenbendazole (50 mg/kg per os q24) associated with pyrantel pamoate and praziquantel for three consecutive days.

These results confirmed the occurrence of *A. abstrusus* infection in cats from Vilhena. The prevalence observed in the population evaluated (1.98%) was similar to that reported in Mato Grosso (1.3%) (Ramos et al., 2013), and Acre (2.5%) (Lima et al., 2021). This is the third record of *A. abstrusus* in domestic cats in northern Brazil (Lima et al., 2021).

The data obtained in this study indicated that most owners in the municipality of Vilhena chose outdoor management for their cats and were often unable to collect cat feces. Accordingly, the prevalence detected in this study could have underestimated the true occurrence of *A. abstrusus* in the investigated area.

Owing to the low positivity obtained in this study, it was not possible to determine the risk factors for infection. However, more than half of the owners (68.32%) detected the presence of intermediate hosts in the cat environment. Cats living in rural areas with outdoor access were particularly susceptible to the disease compared to cats from urban areas (Mircean et al., 2010). The correlation between infection and lifestyle is relevant, since cats with an outdoor lifestyle are at a greater risk of coming into contact with intermediate or paratenic hosts (Beugnet et al., 2014).

Although the Baermann technique is considered the gold standard for diagnosing aelurostrongylosis (Alho et al., 2013), Cat 2 scored positive only for the Hoffman technique possibly due to low parasitism once a single L1 specimen was found.

To overcome Baermann sensivity in the diagnosis of aelurostrongylosis, other diagnostic techniques can be used, such as polymerase chain reaction, although they are not commercially available in Brazil (Lima et al., 2021).

Regarding hematological alterations, Cat 1 had mild neutropenia and eosinophilia, which might have been related to the presence of *A. abstrusus*. Eosinophilia and leukocytosis were previously detected in six cats that were experimentally infected with *A. abstrusus.* Mild anemia, occasional lymphocytosis, monocytosis, and basophilia are non-specific and cannot confirm the diagnostic suspicion of aelurostrongylosis (Schnyder et al., 2014).

Although Cat 1 was subclinically infected, radiographic alterations of the thorax were observed (mild pneumothorax, lung hyperinflation, and mild bronchial pattern). Cats can be clinically healthy despite the presence of radiographic lesions. However, thoracic radiographic findings of aelurostrongylosis are not specific and may be similar to those found in allergic respiratory diseases, as radiographs often demonstrate an interstitial pattern, although the alveolar pattern predominates during the period of more intense larval excretion (approximately 5-15 weeks after infection) (Traversa & Di Cesare, 2016).

Currently, there are several treatment options for aelurostrongylosis, including fenbendazole (Lima et al., 2021), moxidectin/imidacloprid, milbemycin oxime, emodepside, selamectin, eprinomectin (Traversa & Di Cesare, 2016), fipronil/(S)-methoprene/eprinomectin/praziquantel (Giannelli et al., 2015) and moxidectin/fluralaner (Raue et al., 2021).

In this study, Cat 1 was introduced into the shelter without prior deworming and quarantine, which could have spread the L1 in the environment, acting as a source of infection for intermediate (Diakou et al., 2015).

This study confirms the occurrence of *A. abstrusus* in 1.98% of the examined cats from Vilhena, Rondônia. The feline population is at risk of infection, underlining the importance of constant epidemiologic surveillance. Considering the potential clinical impact of *A. abstrusus* in cats, it is advisable to include aelurostrongylosis in the differential diagnosis of respiratory diseases in cats.
